# Chinese Yellow Rice Wine Processing with Reduced Ethyl Carbamate Formation by Deleting Transcriptional Regulator Dal80p in *Saccharomyces cerevisiae*

**DOI:** 10.3390/molecules25163580

**Published:** 2020-08-06

**Authors:** Tianyu Wei, Zhihua Jiao, Jingjin Hu, Hanghang Lou, Qihe Chen

**Affiliations:** Department of Food Science and Nutrition, Zhejiang University, Hangzhou 310058, China; 21913067@zju.edu.cn (T.W.); vi19971215@126.com (Z.J.); y98113@yeah.net (J.H.); louhanghang@zju.edu.cn (H.L.)

**Keywords:** ethyl carbamate, Dal80p, nitrogen catabolite repression, Chinese rice wine brewing

## Abstract

Ethyl carbamate (EC) is a potential carcinogen that forms spontaneously during Chinese rice wine fermentation. The primary precursor for EC formation is urea, which originates from both external sources and arginine degradation. Urea degradation is suppressed by nitrogen catabolite repression (NCR) in *Saccharomyces cerevisiae*. The regulation of NCR is mediated by two positive regulators (Gln3p, Gat1p/Nil1p) and two negative regulators (Dal80p/Uga43p, Deh1p/Nil2p/GZF3p). *DAL80* revealed higher transcriptional level when yeast cells were cultivated under nitrogen-limited conditions. In this study, when *DAL80*-deleted yeast cells were compared to wild-type BY4741 cells, less urea was accumulated, and genes involved in urea utilization were up-regulated. Furthermore, Chinese rice wine fermentation was conducted using dal80Δ cells; the concentrations of urea and EC were both reduced when compared to the BY4741 and traditional fermentation starter. The findings of this work indicated Dal80p is involved in EC formation possibly through regulating urea metabolism and may be used as the potential target for EC reduction.

## 1. Introduction

Ethyl carbamate (EC) is a multisite carcinogen described with effects in experimental animals. It was upgraded to Group 2A by the International Agency for Research on Cancer (IARC) in 2007, indicating that it is a probable carcinogen to human health [1]. EC has been reported to be widespread in fermented foods and beverages, which has raised great health concerns. Indeed, several studies have shown that brandy, sake, and sugar cane spirit contained high levels of EC [2,3,4]. Moreover, an investigation revealed that Chinese rice wine contains higher levels of EC than other alcoholic beverages [5]. 

Several metabolic pathways have been identified for EC formation. The major precursors participating in the formation of EC include carbamyl groups, such as urea, citrulline, and carbamoyl phosphate [6]. The reaction between urea and ethanol is the most common pathway of EC formation. Urea derived from both its abundance in rice and its accumulation from arginine catabolism [2]. Currently, convincing evidence has demonstrated that nitrogen catabolism repression (NCR) governs the utilization of arginine and urea, which indirectly leads to the formation of EC [7]. As a transcriptional control system, NCR imparts a hierarchy to nitrogen sources. Specifically, *Saccharomyces cerevisiae* preferentially selects good nitrogen sources to support cell growth, such as glutamine, asparagine, and ammonium, while it exerts transcriptional repression on the utilization of other poor nitrogen sources, such as arginine, citrulline, and urea [8,9]. During the typical wine fermentation, yeast cells prefer to use ammonium at the initial stage and then suppress the utilization of other nitrogen sources such as arginine. When the preferred ammonium is consumed up, yeast cells shift to use arginine as the predominant nitrogen source, while suppressing the degradation of urea [7,10,11].

It is well known that four transcriptional regulators (Dal80p, Gzf3p/Nil2p, Gln3p, and Gat1p/Nil1p) contribute to the NCR regulation system [12]. Dal80p and Gzf3p/Nil2p act as repressors under poor and preferred nitrogen conditions, respectively, while Gln3p and Gat1p/Nil1p function as positive activators under these conditions [13]. Gln3p and Gat1p have been thoroughly investigated for their abilities to regulate urea and EC accumulation, the mutation of phosphorylation sites on the nuclear localization signal of Gln3p was found to relieve its ability to activate *DUR1,2*. The accumulation of EC decreased in model Chinese rice wine system when fermented with the mutant strains [7]. Moreover, the modifications of Gat1p (mutations on the nuclear localization signals and truncation on the nuclear localization regulatory signals) significantly increased the utilization of non-preferred nitrogen including urea, proline, and allantoine [14]. In our previous study, we analyzed the genes with up-regulated expression levels when yeast cells were cultured under non-preferred nitrogen conditions, and found Dal80p was the most likely candidate for suppression of urea metabolism [15]. Additionally, a series of experiments provided key insights into the properties of Dal80p and its regulation of urea and arginine metabolism. Specifically, the transcription of *DAL80* was sensitive to NCR by itself [13]. Moreover, the disruption of the *DAL80* gene demonstrated that *DUR1,2* was highly expressed, even without an inducer [16]. Additionally, it was reported that the deletion of *DAL80* had no significant effect on *CAR1* (encoding arginase) expression [16]. 

Therefore, we conducted the present experiments to investigate the following hypothesis: during the late stage of Chinese rice wine fermentation, yeast cells shift to poor nitrogen conditions and start to use arginine as the primary nitrogen source, after which the expression of *DAL80* is released from NCR regulation and it is highly expressed. The high expression of Dal80p has no effect on arginine metabolism, while it exerts inhibition on urea degradation. This contributes to urea and EC accumulation during the late fermentation stage. To evaluate this hypothesis, we used a dal80Δ mutant to investigate the effect of *DAL80* deletion on urea and arginine metabolism. Then, the dal80Δ mutant was practically used in Chinese rice wine brewing to check its potential to mitigate EC formation. 

## 2. Results and Discussion

### 2.1. Effect of DAL80 Deletion on Cell Growth, Intracellular Arginase Activity and Urease Activity

**Figure 1:**
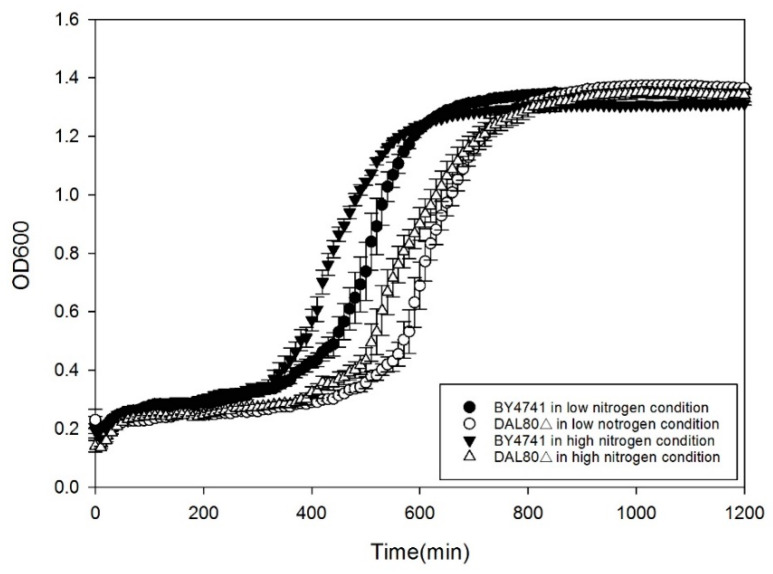
Growth curves of BY4741 and dal80Δ strains under poor nitrogen condition and rich nitrogen condition.

**Figure 2:**
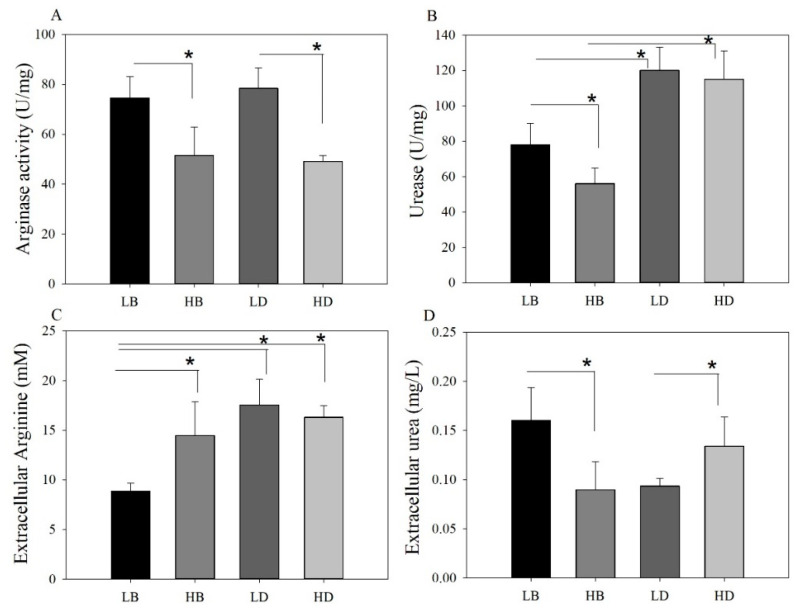
Effect of *DAL80* deletion on the activity of arginase and urease enzymes, as well as the effect on arginine and the extracellular urea concentration when yeast cells were cultivated under poor nitrogen conditions and rich nitrogen conditions. (**A**) Comparison of arginase activity when BY4741 and dal80Δ were cultivated under low nitrogen conditions and high nitrogen conditions (OD600 0.80). (**B**) Comparison of urease activity when BY4741 and dal80Δ were cultivated under poor nitrogen conditions and rich nitrogen conditions (OD600 0.80). (**C**) Comparison of extracellular arginine concentration when BY4741 and dal80Δ were cultivated under low nitrogen conditions and high nitrogen conditions (OD600 0.80). (**D**) Comparison of extracellular urea concentration when BY4741 and dal80Δ were cultivated under low nitrogen conditions and high nitrogen conditions (OD600 0.80). LB: BY4741 under low nitrogen conditions. HB: BY4741 under high nitrogen conditions. LD: dal80Δ under low nitrogen conditions. HD: dal80Δ under high nitrogen conditions. Statistical analysis was performed using one-way analysis of variance (ANOVA) with post-hoc Tukey HSD test (mean ± SD, n = 3, * *p* < 0.05).

**Figure 3:**
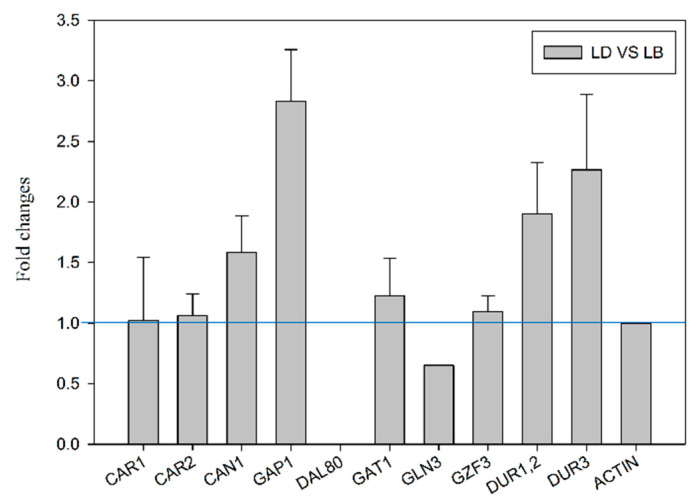
Effect of *DAL80* deletion on the transcriptional level of non-preferred nitrogen metabolic genes as well as the NCR regulatory genes. LB: BY4741 under poor nitrogen conditions. LD: dal80Δ under poor nitrogen conditions.

In the previous work, comparative transcriptome strategy was conducted to identify the major factor affecting EC metabolism by *S. cerevisiae* under high nitrogen and low nitrogen conditions; data show that transcriptional factor *DAL80p* is the most important under two kinds of nitrogen conditions [15]). The different regulatory effect on *DUR1,2* and *CAR1* by Dal80p reported previously prompted us to ask whether the deletion of *DAL80* would remove its regulation of urea degradation. To address this issue, the wild-type (BY4741) and dal80Δ were cultured axenically in two kinds of synthetic defined media (SD1 and SD2). We then analyzed arginase activity, urease activity, and changes in the concentration of arginine and urea. Firstly, we investigated whether the removal of DAL80 impacted yeast cell growth. As shown in Figure 1, we observed dal80Δ cells grew slower than the wild-type yeast. Additionally, both BY4741 and dal80Δ grew slower in poor nitrogen conditions when compared to them growing in rich nitrogen conditions. Figure 2A demonstrated that BY4741 exhibited significantly higher arginase activity under low nitrogen conditions than BY4741 under high nitrogen conditions (LB: BY4741 under low nitrogen conditions. HB: BY4741 under high nitrogen conditions, LB vs. HB). Dal80Δ was the same as BY4741, while arginase activity under low nitrogen conditions was much higher than that under high nitrogen conditions (LD: dal80Δ under low nitrogen conditions. HD: dal80Δ under high nitrogen conditions, LD vs. HD). However, there was insignificant difference between BY4741 and dal80Δ (LB vs. LD, HB vs. HD). Urease activity in BY4741 was completely different from that in dal80Δ (Figure 3); specifically, dal80Δ showed a much higher level of urease activity under both low and high nitrogen conditions than BY4741 (LB vs. LD, HB vs. HD). Furthermore, BY4741 showed higher urease activity under poor nitrogen conditions than that under rich nitrogen conditions (LB vs. HB). Moreover, dal80Δ had high urease activity, which showed no difference between low nitrogen conditions and high nitrogen conditions. Under poor nitrogen conditions, both BY4741 and dal80Δ shifted to use arginine as the main nitrogen source, which results in a higher arginase activity than that under high nitrogen conditions. *DAL80* deletion did not affect arginase activity. However, dal80Δ conferred higher urease activity under both poor and rich nitrogen conditions than BY4741. These results showed that dal80p may negatively regulate urease activity. 

### 2.2. Comparative Analysis of Urea and Arginine Metabolism

As for BY4741, the extracellular arginine concentration under low nitrogen conditions was lower than that under high nitrogen conditions (LB vs. HB), which is due to that BY4741 under poor nitrogen conditions showed higher arginase activity. However, for dal80Δ strain, the concentration of arginine under both low and high nitrogen conditions was higher than BY4741 under low nitrogen conditions (LB vs. LD, LB vs. HD) (Figure 2). Together with the following findings in Reverse Transcription- RealTime Quantitative Polymerase Chain Reaction (RT-qPCR) experiments, we supposed that *DAL80* deletion up-regulated some genes involved in arginine formation or transport. In addition, urea assays revealed that BY4741 under low nitrogen conditions showed an increased level of extracellular urea concentration than that under high nitrogen conditions (LB vs. HB). This was on account of high arginase activity, which formed more urea and suppressed urea degradation. While dal80Δ is opposite to BY4741, dal80Δ under high nitrogen conditions had more extracellular urea than that under low nitrogen conditions (LD vs. HD) (Figure 2). *DAL80* deletion may remove the suppression of urea degradation and enable cells to accumulate less urea under low nitrogen conditions. 

### 2.3. Transcriptional Analysis of Genes Related to Arginine and Urea Metabolism

Moreover, we performed RT-qPCR to analyze the effects of *DAL80* deletion on gene expression. Both BY4741 and dal80Δ were cultivated under poor nitrogen conditions, after which the transcriptional profiles of 10 genes related to arginine and urea metabolism in yeast were examined (Figure 3). Four genes, including *GAP1* (encoding general amino acid permease), *CAN1* (encoding arginine permease), *DUR1,2*, and *DUR3* (encoding urea permease), showed up-regulated expression level in dal80Δ when compared to BY4741. Specially, *GAP1* and *CAN1* were up-regulated, which contributed to the high arginine concentration in dal80Δ (Figure 3). However, the genes associated with arginine degradation (*CAR1* and *CAR2*) and another three NCR regulators (*GAT1*, *GLN3,* and *GZF3*) showed an insignificant difference.

### 2.4. The Genetic Strain with the Modification of DAL80 Can Reduce Urea Accumulation during Chinese Rice Wine Fermentation

Urea is the most dominant EC precursor; therefore, strategies that could contribute to the mitigation of urea accumulation could be applied to decrease EC formation in Chinese rice wine fermentation [15,17]. These results drove us to conduct rice wine brewing with the *S. cerevisiae* dal80Δ and test the effects of *DAL80* deletion on EC formation. In considering that the genome sequence for *S. cerevisiae* that was isolated from Chinese rice wine exhibited high similarity with BY4741 [18], a brew with a traditional starter or *S. cerevisiae* BY4741 was used as two of the control samples; a mixture that was made of half traditional fermentation starter and half *S. cerevisiae* dal80Δ was also included to fully assess the effects of *DAL80* modification. 

**Figure 4:**
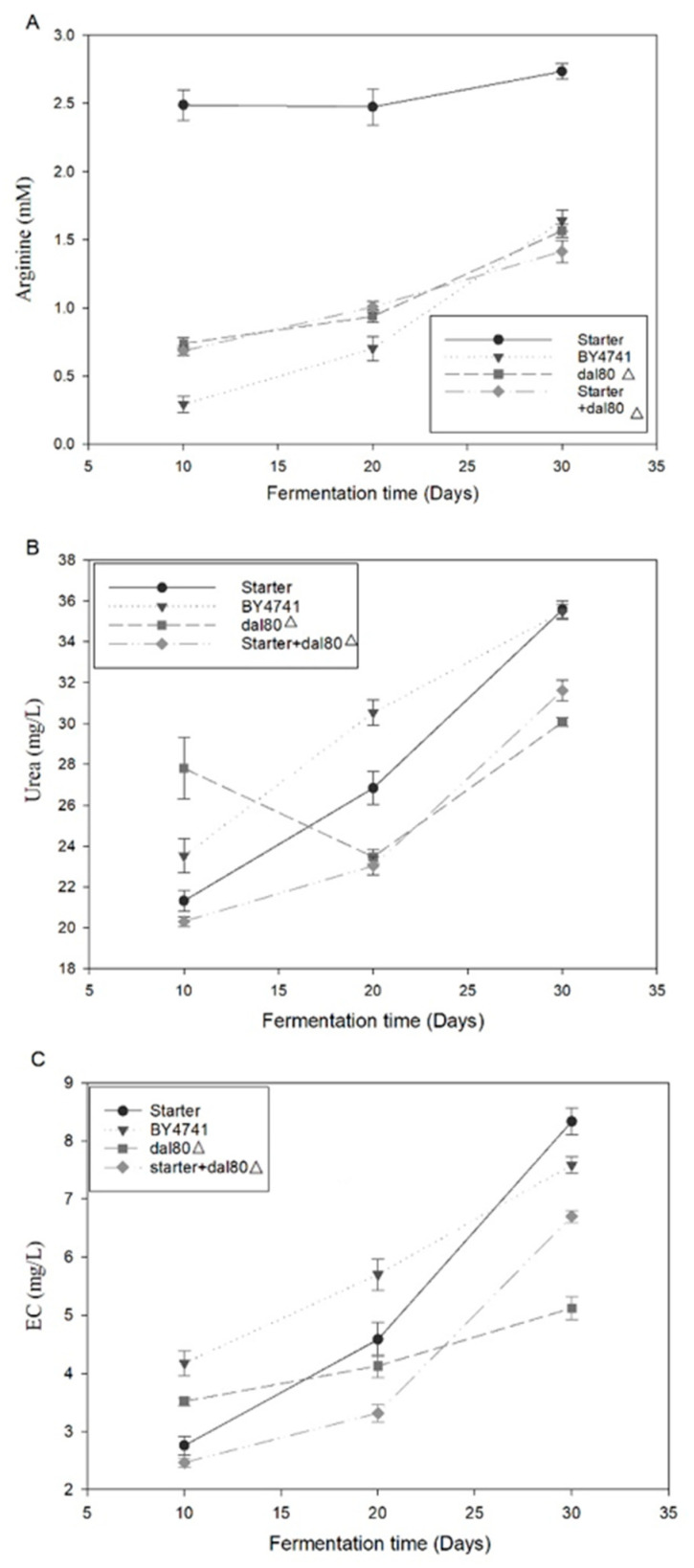
Effect of *DAL80* deletion on EC formation during the process of Chinese rice wine fermentation. (**A**) Comparison of arginine during the process of fermentation with starter, BY4741, dal80Δ, and a mixture of fermentation starter (50%) and dal80Δ (50%). (**B**) Comparison of urea during the process of fermentation with starter, BY4741, dal80Δ, and a mixture of starter (50%) and dal80Δ (50%). (**C**) Comparison of EC during the process of fermentation with fermentation strains, such as BY4741, DAL80-deleted yeast (dal80Δ), and a mixture of fermentation starter (50%) and dal80Δ (50%).

We conducted Chinese rice wine brewing using the *S. cerevisiae* dal80Δ strain and tested the effects of *DAL80* deletion on EC formation. The brewing broth was collected every 10 days for 30 days and the levels of arginine, urea, and EC were assayed. As shown in Figure 4A, the brewing process with the traditional fermentation starter resulted in a much higher arginine concentration than the other samples; the high level of extracellular arginine resulted from the diverse microbes existed in the fermentation starter. On the other hand, the difference in extracellular arginine concentration among BY4741, dal80Δ, and the mixture of dal80Δ and fermentation starter was slight. These findings indicated that *DAL80* deletion likely has an insignificant effect on arginine metabolism. Combining the above data and the previous report of transcriptional study, we supposed that the *DAL80* gene may influence urea metabolism. 

To further elucidate the effect of *DAL80* gene in yeast cell on urea metabolism, we present the data shown in Figure 4B. All four groups showed increasing urea concentration throughout the fermentation process. The urea concentration is determined by both arginine degradation and urea catabolism. The increasing urea concentration during the fermentation process accounted for the accumulation of arginine degradation and suppression of urea catabolism. After 30 days of fermentation, the brewing with traditional starter and BY4741 generated more urea than dal80Δ, while the difference between dal80Δ and its mixture with starter was slight (Figure 4B). These findings imply that dal80Δ cells might play a crucial role when fermented with the mixture of dal80Δ and starter.

### 2.5. DAL80 Deletion Can Decrease EC Formation during Chinese Rice Wine Fermentation

EC is spontaneously formed by the reaction between ethanol and urea [19]; therefore, EC may positively correlate with urea. Figure 4C showed that EC concentration increased throughout the fermentation process as urea did, these results were consistent with the reports by Zhao [7]. In this work, after 30 days of fermentation, the brewing process with traditional fermentation starter generated the highest level of EC, followed by brewing with BY4741 and the mixture of dal80Δ and traditional fermentation starter. The fermentation with dal80Δ led to the lowest level of EC formation (Figure 4C). Fermentation with traditional starter generated the highest level of EC, which was in accordance with the findings by Fang et al. [20]. We reasoned that the *lactic acid bacteria* (LAB) existed in the starter may contribute to the EC formation [21,22]. Meanwhile, the compositions and concentration of amino acids are primary indicators of Chinese rice wine quality [20]. As presented in Table 1, the concentration of major amino acids differed slightly. The deletion of *DAL80* gene had a significant impact on ethanol metabolism in *S. cerevisiae*, but the fermentation mixture of starter and dal80Δ resulted in higher ethanol concentration, which needs further investigation. 

## 3. Materials and Methods

### 3.1. Strains, Plasmids, and Culture Conditions

*Saccharomyces cerevisiae* BY4741 (MATa, his3Δ1, leu2Δ0, met15Δ0, ura3Δ0) and dal80Δ (BY4741 DAL80::KanMX4) were obtained from the European Saccharomyces cerevisiae Archive for Functional Analysis (EUROSCARF)(Scientific Research and Development GmbH, Köhlerweg, Germany). The traditional brewing starter with mixed microbes was originated from Wuzhanmao Rice Wine Making Ltd., Zhejiang, China. The preparation *S. cerevisiae* BY4741 and the knocked-out strains were conducted in a 5-L fermenter for large-scale fermentation at 28 °C. The cultured yeast cells were washed and sterilely frozen for further use.

The yeast strains were cultured on Yeast Extract Peptone Dextrose Medium (YPD or YEPD):10 g/L yeast extract, 20 g/L peptone, 20 g/L glucose, 2% agar). Synthetic defined medium SD1, which was composed of YNB medium (1.7 g/L yeast nitrogen base with no ammonium sulfate and amino acids), 20 g/L glucose, 2 mM ammonium sulfate, and 100 mL/L 10× dropout mix (200 mg/L L-arginine monohydrochloride, 200 mg/L histidine, 1000 mg/L leucine, 200 mg/L methionine, and 200 mg/L uracil) was used to mimic the nitrogen conditions during the late stage of Chinese rice wine fermentation. Synthetic defined medium SD2, which contained 1.7 g/L YNB medium, 20 g/L glucose, 40 mM ammonium sulfate, and 100 mL/L 10× dropout mix, was used as a control medium with rich nitrogen. 

### 3.2. Yeast Cell Growth Curve Assay

BY4741 and dal80Δ cells were grown on YPD agar, after which they were shaken in YPD medium until an optical density at a wavelength of 600 nm (OD600) value of 0.1 was attained. The cultured cells were then collected by centrifugation at 3000× *g* for 5 min, washed twice with targeted medium (SD1 or SD2) and resuspended. Four samples (BY4741 in SD1, BY4741 in SD2; dal80Δ in SD1, dal80Δ in SD2) were subjected to serial two-fold dilution and then cultivated in 96-well plates. During cultivation period, OD600 value was recorded every 10 min to develop the growth curve. Growth curve assays for each group were conducted in triplicate.

### 3.3. Reverse-Transcribed Quantitative PCR (RT-qPCR) Analysis

BY4741 and dal80Δ were cultured under poor nitrogen conditions (SD1 medium) until OD600 value reached 0.8. Total RNA was then extracted with a yeast RNA kit (Omega Bio-tek, Inc. Norcross, GA, USA), after which we analyzed RNA purity and concentration using a Nanodrop 2000 spectrophotometer (Thermo Scientific, Wilmington, DE, USA). Before synthesizing cDNA, genomic DNA was eliminated by adding gDNA wiper mix (Vazyme, Nanjing, China). RNA was then reverse-transcribed using HiScript^®^ QRT SuperMix for qPCR (Vazyme, Nanjing, China).

The primers for RT-qPCR are listed in Table S1. qPCR reaction was performed with ChamQ SYBR qPCR Master Mix (Vazyme, Nanjing, China). qPCR was run by subjecting samples to 40 cycles of 95 °C for 10 s and 60 °C for 30 s. The specificities of the primers were then examined by determining melting curves by subjecting samples to 95 °C for 15 s, 60 °C for 60 s, and 95 °C for 15 s. The fold changes were analyzed using 2^−ΔΔCt^ [23], where Ct is the threshold cycle. The actin gene was used as a standard. RNA preparation and qPCR for each group were conducted in triplicate.

### 3.4. Shaking-Flask Cultivation

BY4741 and dal80Δ strains were incubated in SD1 and SD2 fermentation medium at 30 °C with continual shaking until the OD600 value reached 0.8. The samples were then subjected to assays of extracellular arginine concentration, extracellular urea concentration, intracellular arginase activity, and intracellular urease activity. All the assays were performed in triplicate.

### 3.5. Chinese Yellow Rice Wine Fermentation with Genetically Modified Strain

Fermentation was conducted according to the process for manufacturing of Chinese yellow rice wine (20). In this work, there were four groups as shown in Table 1. During the fermentation process, samples were collected for assays of urea, arginine, and EC concentration at three-time points (10 days, 20 days, 30 days). After 30 days fermentation, the remaining samples were filtered and boiled (90 °C, 20 min), after which the levels of ethanol, amino acids, and flavor compounds were examined. Each group was conducted in triplicate.

### 3.6. Assay of Arginase Activity, Urease Activity, Urea, Arginine, EC, Ethanol, and Amino Acids

For assays of arginase and urease activities, yeast cells were harvested by centrifugation 3000× *g* (4 °C, 5 min), after which crude protein was extracted using a one-step yeast active protein extraction kit (Sangon Biotech, Shanghai, China). The supernatant of cell extracts was then collected for the following assays. Total protein was determined by the method described by Bradford [24]. Arginase activity was detected using a QuantiChrom^TM^ Arginase Assay Kit (BioAssay Systems, Hayward, CA, USA). One unit of enzyme was defined as the amount of enzyme that catalyzed 1 μmol/L of L-arginine to ornithine and urea per minute at pH 9.5 and 30 °C. Urease activity was examined with a QuantiChromTM Urease Assay Kit (BioAssay Systems, Hayward, CA, USA). One unit of enzyme was defined as the amount of enzyme that catalyzed urea to 1 μmol ammonia per minute at pH 7.4 and 30 °C.

Standards of L-arginine and urea were obtained from Sangon Biotech Corporation (Shanghai, China). EC and 9-xanthydrol were bought from Sigma-Aldrich Chemical (Saint Louis, MO, USA). To determine urea concentration, high-performance liquid chromatography (HPLC) coupled with fluorescence detection and automated derivatization with xanthydrol was used. The detailed procedure was conducted as described in the previous report [25]. Arginine concentration was quantified using HPLC and pre-column derivatization with 3-MPA/OPA and FMOC-Cl [26]. The analysis of EC was based on a previous study conducted by our lab [27]. Ethanol was determined with an alcohol meter as mentioned in the previous literature [28]. The composition and concentration of amino acids were examined using an automatic amino acid analyzer (L-8900; Hitachi company, Tokyo, Japan).

## 4. Conclusions

**Figure 5:**
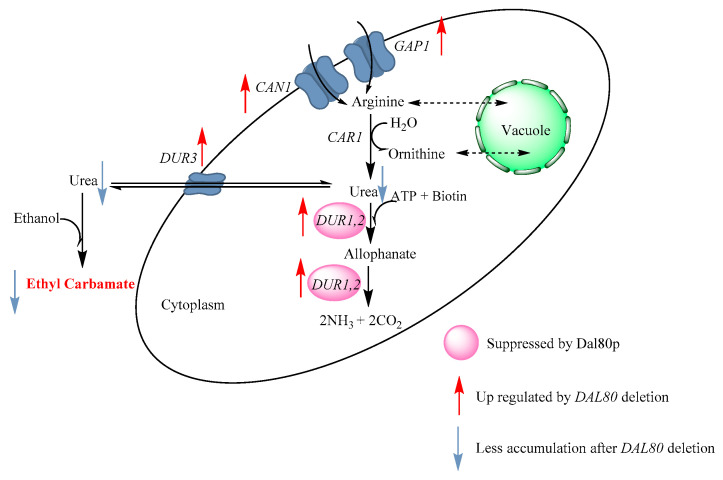
The proposed regulation pathway of arginine and urea metabolism by GATA factor Dal80p in *S. cerevisiae*.

As can be seen in Figure 5, in this work we found that *DAL80* was activated upon yeast cells shifted to consume arginine as the main nitrogen source at the late stage of fermentation, which generally results in the suppression of urea degradation. Urea accumulation leads to the increased formation of EC during Chinese rice wine fermentation. *DAL80* deletion may promote urea degradation without affecting arginine metabolism; this provide the key foundation to apply dal80Δ in the Chinese rice wine system to decrease EC formation without affecting arginine utilization. Additionally, the genes related to urea degradation (*DUR1,2* and *DUR3*) and arginine transport (*GAP1* and *CAN1*) showed higher expression level in Dal80Δ under the nitrogen catabolite repression environment. Specifically, it is possible that blocking DUR3 can stop urea secretion, which leads to a very low production of EC. However, the interconnection of DUR3, DUR1,2, and other genes may interfere the effect. After that, the use of dal80Δ in the model rice wine system generated the decreased EC accumulation, and had little effect on the composition and concentration of amino acids. Our findings led to the conclusion that Dal80p is involved in EC formation through the regulation of urea metabolism by *S. cerevisiae* during Chinese yellow rice brewing. However, it is still needed to conduct more research focusing on the correlation between *DAL80* and arginine metabolism network at the systematic level. 

## Figures and Tables

**Table 1:** Measurement of ethanol and amino acids in the fermentation of Chinese rice wine ^1^.

Compound	Starter	BY4741	dal80Δ	Starter + dal80Δ
Ethanol (% vol)	11.90 ± 0.20	11.40 ± 0.30	10.20 ± 0.30	13.40 ± 0.50
Amino acids (mg/liter)				
Asparagine	125.32 ± 0.56	124.65 ± 0.56	134.32 ± 0.46	114.32 ± 0.53
Threonine	60.23 ± 0.32	54.65 ± 0.16	56.98 ± 0.52	58.65 ± 0.14
Serine	112.32 ± 0.71	116.65 ± 0.76	115.65 ± 0.33	130.32 ± 0.58
Glutamate	169.12 ± 0.78	145.65 ± 0.58	169.32 ± 0.54	164.32 ± 0.62
Glycine	150.32 ± 0.64	154.32 ± 0.46	171.32 ± 0.5	162.32 ± 0.27
Alanine	333.32 ± 0.93	352.32 ± 1.46	361.06 ± 0.41	345.32 ± 0.74
Cystine	100.32 ± 0.55	114.65 ± 0.66	133.32 ± 0.52	129.32 ± 0.53
Valine	127.65 ± 0.56	134.65 ± 0.44	140.65 ± 0.26	139.32 ± 0.25
Methionine	9.32 ± 0.06	10.65 ± 0.02	9.65 ± 0.02	11.65 ± 0.62
Isoleucine	90.32 ± 0.26	100.32 ± 0.40	95.65 ± 0.63	97.65 ± 0.27
Leucine	216.32 ± 0.66	241.65 ± 0.52	236.3 ± 0.62	244.32 ± 0.36
Tyrosine	215.65 ± 0.98	245.23 ± 0.83	236.32 ± 0.73	242.32 ± 0.17
Phenylalanine	216.32 ± 0.86	231.65 ± 0.47	198.96 ± 0.71	200.65 ± 0.62
Lysine	154.65 ± 0.56	188.32 ± 0.82	159.65 ± 0.26	184.32 ± 0.25
Histidine	90.65 ± 0.12	103.65 ± 0.43	99.46 ± 0.29	100.65 ± 0.74
Arginine	399.98 ± 0.76	395.32 ± 0.23	397.32 ± 0.66	421.32 ± 0.76
Proline	245.32 ± 0.53	288.32 ± 0.52	245.65 ± 0.73	253.32 ± 0.45

^1^ Four groups were designed for Chinese rice wine fermentation. In the starter group, 0.5 kg sticky rice, 0.6 L H_2_O, 90 g wheat Qu, and 1 g Chinese fermentation starter were added. In the BY4741 group, 0.5 kg sticky rice, 0.6 L H_2_O, 90 g wheat Qu, and 1 g BY4741 were added. In the dal80Δ group, 0.5 kg sticky rice, 0.6 L H_2_O, 90 g wheat Qu, and 1 g dal80Δ were added. In the fermentation starter + dal80Δ group, 0.5 kg sticky rice, 0.6 L H_2_O, 90 g wheat Qu, 0.5 g dal80Δ, and 0.5 g Chinese fermentation starter were added. These data were assayed at the end of the fermentation. Data are expressed as means ± SD of the assays. Determinations were made in duplicated.

## Supplementary Materials

The following are available online. Table S1: ligonucleotides for RT-qPCR.
