# Supplementary material for: Chinese Yellow Rice Wine Processing with Reduced Ethyl Carbamate Formation by Deleting Transcriptional Regulator Dal80p in Saccharomyces cerevisiae
Source: Molecules. 2020 Aug 6;25(16):3580. doi: 10.3390/molecules25163580 (PMC7464398; doi:10.3390/molecules25163580)
Supplement: Supplementary file 1 [file molecules-25-03580-s001.pdf]

Supplementary Materials

Tianyu Wei, Zhihua Jiao, Jingjin Hu, Hanghang Lou and Qihe Chen *

Department of Food Science and Nutrition, Zhejiang University, Hangzhou 310058, China; 21913067@zju.edu.cn (T.W.); vi19971215@126.com (Z.J.); y98113@yeah.net (J.H.); louhanghang@zju.edu.cn (H.L.)

* Correspondence: chenqh@zju.edu.cn; Tel.: +85-571-86984316

Table S1. ligonucleotides for RT-qPCR.

Target genes	Directions [a]	Sequence (5′-3′)
CAR1	F	AATACGGCATCAACGCTGTCATTG
	R	CCACCTCTCACTGGAGTACCTGTA
CAR2	F	CTGGGTGCCGAGGGTAACTT
	R	TTCTGCGTGGCCGTATCTG
GAP1	F	TGGTGGTCCAACAGGTGGTTACAT
	R	GCAGCGGTGACGAAGACAGAAC
CAN1	F	GGAGGATGGCATAGGTGATGAAGAT
	R	GCGTTGGTCAGAGGTGTGGATAA
DUR1,2	F	GGTGTCCCTATTGCTGTTAAG
	R	CCGTGTGCCGACTAATCC
DUR3	F	ACTGCCTGTGGGTGTTGTTG
	R	CGTCTACTGGATGCCTCTTGG
GLN3	F	AAGACGGTCAAGGACACCACAC
	R	TGTTGGTTGGATGCGGCTATGT
GAT1	F	GGCAGCGTCCAAGAGGAAGAAC
	R	GGCGAGTGTTAGAGCGGAGGTA
GZF3	F	GCATCGCAGGCTACAACTCTTC
	R	GCCATCTCCCAATATGCCCTTC
DAL80	F	GCTTGAAGACGGACACCATTAAGTC
	R	ACCACCACCAGTTGTAAGCAGTC
ACT1	F	TTATTGATAACGGTTCTGGTATG
	R	CCTTGGTGTCTTGGTCTAC

[a] F, forward; R, reverse.
